# A research on the improved rotational robustness for thoracic organ delineation by using joint learning of segmenting spatially‐correlated organs: A U‐net based comparison

**DOI:** 10.1002/acm2.14096

**Published:** 2023-07-19

**Authors:** Jie Zhang, Yiwei Yang, Min Fang, Yujin Xu, Yongling Ji, Ming Chen

**Affiliations:** ^1^ The Cancer Hospital of the University of Chinese Academy of Sciences (Zhejiang Cancer Hospital) Hangzhou Zhejiang China; ^2^ Institute of Basic Medicine and Cancer (IBMC) Chinese Academy of Sciences Hangzhou Zhejiang China

**Keywords:** automatic thoracic organ segmentation, multi‐task learning, related task setting, rotational robustness

## Abstract

**Purpose:**

To study the improved rotational robustness by using joint learning of spatially‐correlated organ segmentation (SCOS) for thoracic organ delineation. The network structure is not our point.

**Methods:**

The SCOS was implemented in a U‐net‐like model (abbr. SCOS‐net) and evaluated on unseen rotated test sets. Two hundred sixty‐seven patients with thoracic tumors (232 without rotation and 35 with rotation) were enrolled. The training and validation images came from 61 randomly chosen unrotated patients. The test data included two sets. One consisted of 3000 slices from the rest 171 unrotated patients. They were rotated by us by –30°∼30°. One was the images from the 35 rotated patients. The lung, heart, and spinal cord were delineated by experienced radiation oncologists and regarded as ground truth. The SCOS‐net was compared with its single‐task learning counterparts, two published multiple learning task settings, and rotation augmentation. Dice, 3 distance metrics (maximum and 95th percentile of Hausdorff distances and average surface distance (ASD)) and the number of cases where ASD = infinity were adopted. We analyzed the results using visualization techniques.

**Results:**

In terms of no augmentation, the SCOS‐net achieves the best lung and spinal cord segmentations and comparable heart delineation. With augmentation, SCOS performs better in some cases.

**Conclusion:**

The proposed SCOS can improve rotational robustness, and is promising in clinical applications for its low network capacity and computational cost.

## 1 INTRODUCTION

Contouring organs at risk (OARs) is a crucial step in clinical radiation treatment. Its accuracy and reproducibility are highly related to tumor control and radiotherapy toxicities.^1^, ^2^, ^3^ A fully convolutional neural network (FCN) has been proven a superior automatic segmentation method.^4^, ^5^, ^6^ However, different immobilization techniques affect FCN's performance, since it likely causes patient rotation in the transverse section and hence leads to an unseen input image during training.

The problem is that the model has not learned well‐generalizing representations, because the supine position is the most common, and less rotated samples exist in the training set. To alleviate it, rotation augmentation^7^, ^8^, ^9^, ^10^, ^11^ is a potential solution. By increasing the size of rotated samples in the training, it helps a model learn how to segment organs from a rotated image. Nalepa et al.^12^ proved that rotation augmentation did boost a model's generalization ability. But the proportion of the numbers of augmented samples at various angles would be a difficult decision, and the expanded training set presents a challenge to the computing hardware. Multi‐task learning (MTL),^13^ a paradigm that learns multiple related tasks jointly, is another way to further exploit labeled data.^14^, ^15^, ^16^, ^17^ The settings of the related tasks are various. An image‐level classification was an auxiliary task in Tao He et al.’s work.^15^ The classification was whether computed tomography (CT) contained the target organs. It helped the segmentation task filter false positive pixels. Similar works were reported by Chakravarty et al.^18^ and Zhou et al.^19^ Shape‐prior, including distance map and contour map, was the complementary tasks in Fernando Navarro et al.’s segmentation report.^20^ Other shape knowledge, such as distinctive curve^21^ and signed distance,^22^ were also adopted. Although these methods improved the network's performance, they increased the capacity of the network's parameters and required more training time. No rotated samples were explicitly reported in their test set. We have no idea of the rotational robustness of these MTLs.

This paper studies improved rotational robustness by using joint learning of spatially‐correlated organ segmentation (SCOS). It is assessed in a U‐net^23^ like architecture for thoracic organ segmentation (i.e., bilateral lung, heart, and spinal cord). The training set solely encompasses CT images scanned in the supine position (i.e., without rotation), but only rotated samples are in the test set.

In our work, the “spatially‐correlated organs” refer to the organs whose locations are close to each other in a human body, such as the neighboring organs in the thorax. SCOS means the tasks of segmenting these organs.

This work consists of four comparisons. The first one is to compare SCOS with its single‐task learning (STL) counterparts as an ablation study. The second one is to compare SCOS with other published settings of multiple tasks for a comprehensive comparison. The third one is to compare SCOS with rotation augmentation. The three comparisons were conducted on the slices rotated by us. The fourth one is to compare the above settings on real rotated cases. Furthermore, we explore the reasons for rotational robustness achieved by SCOS using visualization techniques. Specially, the contributions of the work can be summarized as follows:

1. We study the improved rotational robustness by using joint learning of spatially‐correlated organ segmentations.
2. The proposed SCOS only involves segmentation tasks of spatially correlated organs, rather than other constraints, for a better saving on network capacity and training time.
3. We analyze the results using visualization techniques for FCN interpretation.

To the best of our knowledge, this is the first work to study rotational robustness achieved by SCOS and to explain its improved performance using visualization techniques. The remainder of this paper is organized as follows. In Section 2, we detail the networks of SCOS, its STL counterparts, other comparative MTL configurations, rotation augmentation, and experiment. The results are presented in Section 3 and discussed in Section 4. Finally, we conclude our work in Section 5.

## 2 MATERIALS AND METHODS

### 2.1 Learning task setting of SCOS and its STL counterparts in U‐net like architecture

**FIGURE 1:**
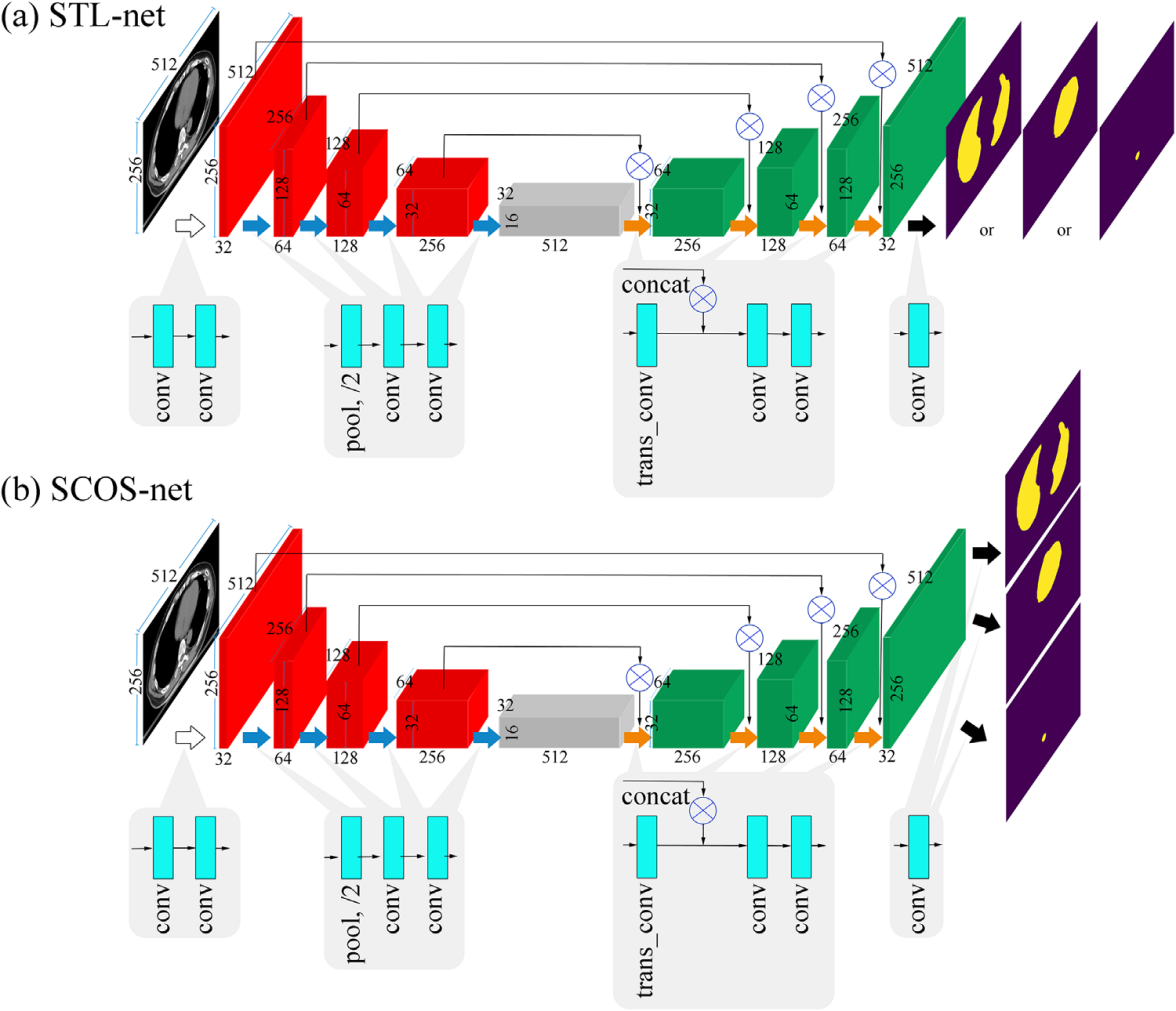
Architectures of SCOS‐net and its single‐task learning counterparts (STL‐net). The white and blue thick arrows indicate encoder blocks. The orange and black thick arrows indicate decoder blocks. The kernel sizes are 1 × 1 and 2 × 2 for the last convolution and the transpose convolution (with the stride of 2 × 2), respectively. Other kernel sizes are 3 × 3. concat, concatenation; conv, convolution; trans_conv, transpose convolution.

Figure 1 illustrates the model details trained with the proposed setting of multiple learning tasks (abbreviated as SCOS‐net) and its STL counterparts (abbreviated as STL‐net).

#### 2.1.1 Details of STL‐net

The STL‐net is open‐source.^24^ Compared to the original U‐net,^23^ the filter number in each layer is reduced by half, as a result of the limited computational ability of the hardware. In the decoding path, the transposed convolution^25^ is adopted, instead of an up‐sampling of the feature map followed by a 2 × 2 convolution. All convolutions in STL‐net are padded ones to guarantee the same size between input and output. All convolution layers use activation functions of ReLU, except the sigmoid in the last one.

#### 2.1.2 Details of SCOS‐net

As exhibited in Figure 1b, SCOS‐net shares the network backbone with STL‐net. At the end of the network, SCOS‐net has three task‐specific convolution layers. They correspond to three OARs segmentation.

### 2.2 Other published multiple task settings in U‐net like architecture

Figure 2 exhibits two published MTL settings for comparison with SCOS‐net. Figure 2a shows the network details of the three methods with a similar structure of SCOS‐net. Figure 2b gives a clear illustration of the different settings of learning tasks. The distance map^20^ and contour map^20^ are auxiliary tasks to help learn the shape representation. The distance map was generated by using the Euclidean distance transform on each organ's segmentation. The contour map was each organ's binary edge.

FIGURE 2(A) Network structures of (a, b) the two comparative task settings and (c) SCOS‐net. The learning task settings are illustrated in (B) for a clear view. (B) Learning task settings of three methods. (a) The proposed SCOS. (b, c) are two comparative ones. Distance map is the cumulative sum of the normalized distance transform of each organ's segmentation. Contour map is each organ's binary edge. concat, concatenation; conv, convolution; trans_conv, transpose convolution

**Figure:**
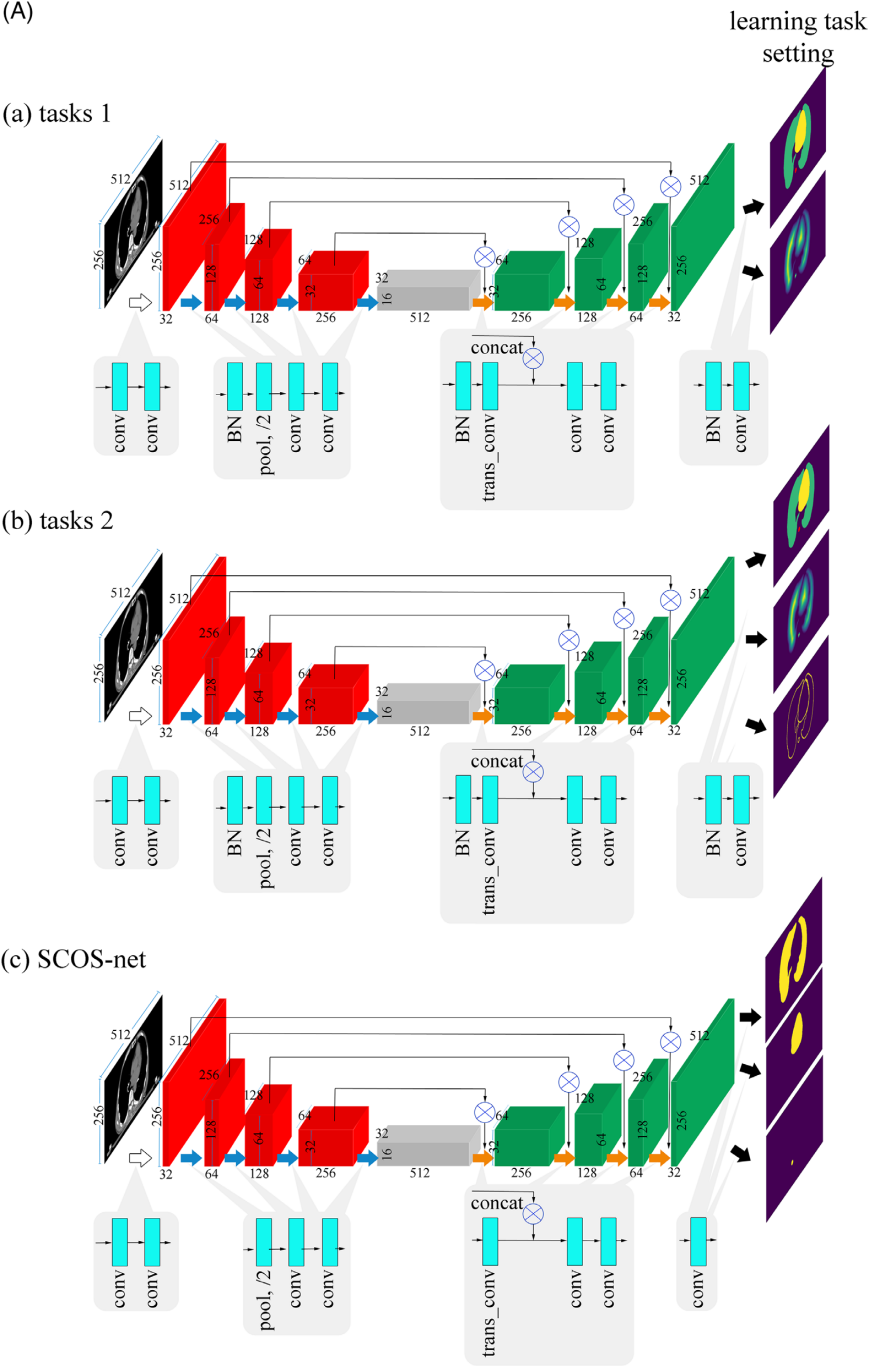

**Figure:**
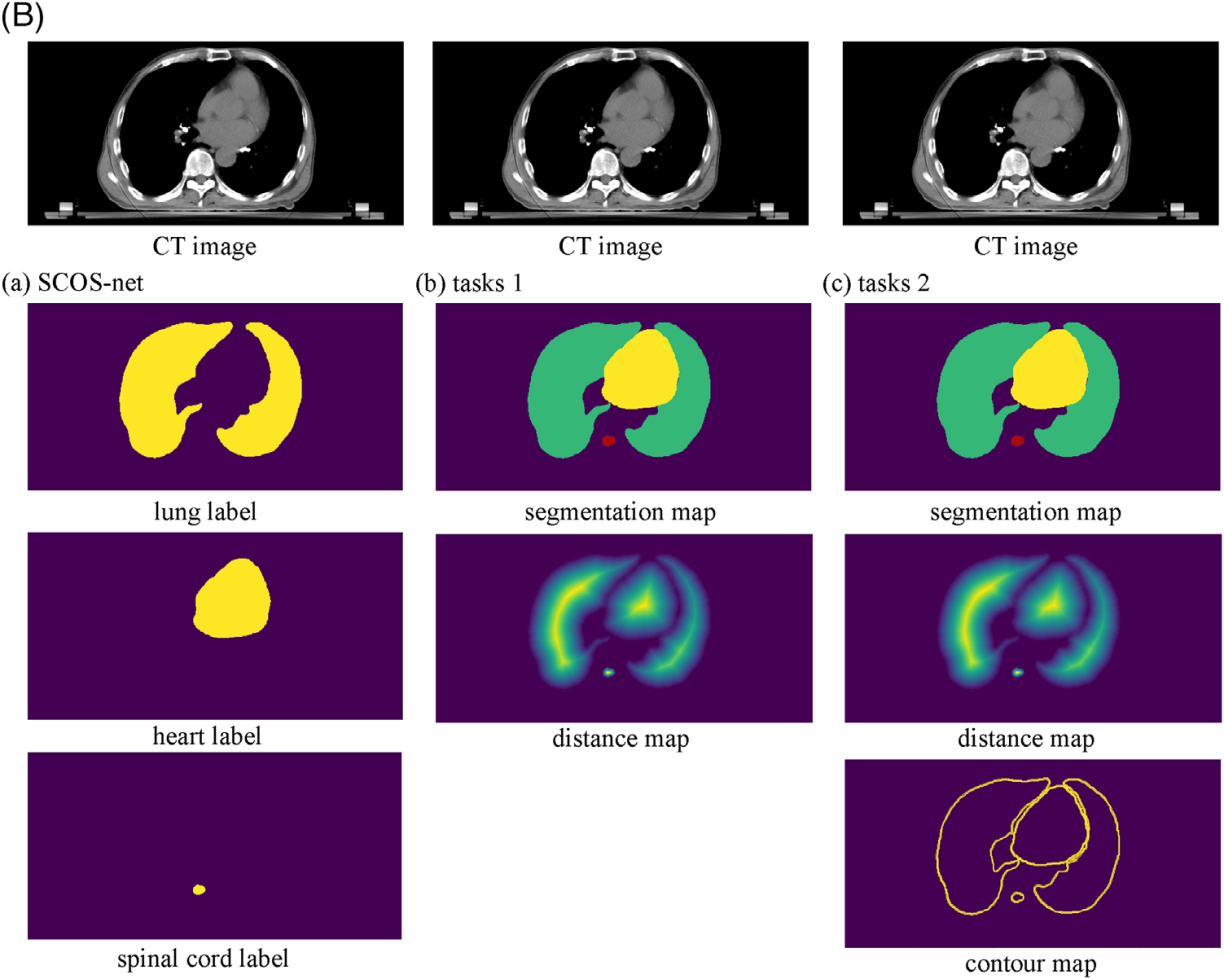

### 2.3 Rotation augmentation with SCOS and its STL counterparts

The SCOS‐net and STL‐net in Figure 1 were trained with randomly‐rotational samples (abbreviated as SCOS_aug_‐net and STL_aug_‐net). Their rotation angle ranged from –30° to 30°.

### 2.4 Experiments

#### 2.4.1 Data acquisition

The CT images were collected from 267 patients with thoracic tumors receiving radiation treatment in our department. Among them, 232 patients underwent CT scan in supine position (i.e., without rotation). The CT slices from the remaining 35 patients showed a rotation ranging from 1.5° to 9.34°.

The CT slices were obtained by a Light Speed (GE Healthcare, Chicago, America) or a Brilliance CT Big Bore system (Philips Healthcare, Best, the Netherlands). They had 512 × 512 pixels with a thickness of 5 mm and a spatial resolution of approximately 1 mm. All OARs were delineated by experienced radiation oncologists and were ground truth in our work. The labeled organs included the lung, heart, and spinal cord.

#### 2.4.2 Image preprocessing

For the sake of saving computing resources, all images were cut into a matrix of 512 × 256 by dropping 512 × 128 pixels from the top and bottom, respectively. The image intensity values were linearly mapped to 0–255 from –135HU ∼215HU.

#### 2.4.3 Data splitting

In the data collected from 267 patients (232 without rotation, 35 with rotation), 1240 CT slices from 61 randomly selected unrotated patients constituted the training set (856 slices) and validation set (384 slices). The test data consisted of two sets.

The first test set was two‐dimension (2D) images rotated by us. It included 3000 slices from the rest of 171 unrotated patients. All experimental images encompassed the lung, heart, and spinal cord. To investigate the robustness against rotation, we applied a random rotation (–30°∼30°) to them and their labels. The second test set was three‐dimension (3D) data from the 35 rotated patients.

The data splitting strategy (i.e., a small amount of training data, but a large amount of test data) is to simulate a real clinical application scenario in which a proposed network is applied on a large number of cases, but trained on a limited quantity of data.

#### 2.4.4 Experiment setup and implementation

We implemented the SCOS‐net, STL‐nets, and other two comparative MTL settings with Python^26^ and used the adaptive moment estimation^27^ (Adam) with a learning rate of 10^−3^, batch size of 8, and epochs of 3000 for training. The training losses (*L*) for the SCOS‐net and STL‐nets were both dice loss functions, as defined in formulas (1) and (2):

(1) $$L_{STL-net}=-\frac{1}{N}\sum_{i=1}^{N}Dice_{i}$$

(2) $$\begin{array}{ccc} & & L_{\mathrm{SC}OS-net}=\\ & & \;-\frac{1}{N}\sum_{i=1}^{N}\left(Dice_{i}^{lung}+Dice_{i}^{heart}+Dice_{i}^{spinalcord}\right) \end{array}$$

(3) $$Dice=\frac{2(X\cap Y)}{X\cup Y}$$

where X was the model result. Y was the ground truth. Dice, ranging from 0 to 1, assessed the overlap between X and Y. *i* meant the *i*th sample. *N* was the number of training data. $L_{SCOS-net}$ was designed to guide SCOS‐net to learn how to simultaneously segment three organs, thus a dice loss for each organ was summed in Equation (2).

The loss functions of the two comparative MTL configurations (indicated as tasks 1 and 2) were

(4) $$L_{tasks1}=L_{seg}+L_{distance}$$

(5) $$L_{tasks2}=L_{seg}+L_{contour}+L_{distance}$$

where *L*_seg_ and *L*_contour_ correlated to segmentation map and contour map respectively. They were both dice loss as defined in formula (2). The distance map related to *L*_distance_:

(6) $$L_{\mathrm{distance}}=\sum_{x}{\left(g(x)-p(x)\right)}^{2}$$

in which *g*(*x*) was the ground truth value of a pixel in location *x*. *p*(*x*) was the predicted one.

#### 2.4.5 Assessment metrics

Five metrics were adopted in this paper: dice, maximum hausdorff distance (maxHD), 95th percentile of Hausdorff distance (95%HD), average surface distance (ASD) and the number of cases with ASD equaling to infinity (*N*(inf)). Dice was defined in formula (3). The distance metrics were calculated as follows:

1. maxHD and 95%HD
(7) $$maxHD=\left\{\begin{array}{c} \max\left(\delta_{H}\left(X,Y\right)\cup \delta_{H}\left(Y,X\right)\right),X\neq \emptyset \\ inf,X=\emptyset \end{array}\right.$$
(8) $$\begin{array}{c} 95\%HD=\left\{\begin{array}{c} \left\{\hat{\delta }|P\left(\delta \leq \hat{\delta }\right)=95\%,\delta \in \left(\delta_{H}\left(X,Y\right)\cup \delta_{H}\left(Y,X\right)\right)\right\},\\ X\neq \emptyset \\ inf,X=\emptyset \end{array}\right. \end{array}$$
  in which
$$\begin{array}{ccc} \delta_{H}\left(X,Y\right) & = & \left\{\min_{y\in Y}\left\|x-y\right\||x\in X\right\},\delta_{H}\left(Y,X\right)\\ & = & \left\{\min_{x\in X}\left\|x-y\right\||y\in Y\right\} \end{array}$$
  X and Y had the same definitions in formula (3). || || was Euclidean distance. $\delta_{H}\left(X,Y\right)$ was a set of distances of all pixels in X to their nearest pixels in Y. $\delta_{H}\left(Y,X\right)$ had the similar meaning, but the distance was from Y to X. Herein, maxHD was the maximum among $\delta_{H}\left(X,Y\right)$ and $\delta_{H}\left(Y,X\right)$. 95%HD was the 95th percentile of all values in $\delta_{H}\left(X,Y\right)$ and $\delta_{H}\left(Y,X\right)$. “inf” meant infinity.
2. ASD
(9) $$ASD=\left\{\begin{array}{c} \frac{1}{n_{X}+n_{Y}}\left(\sum_{i=1}^{n_{Y}}d\left(y_{i},X\right)+\sum_{i=1}^{n_{X}}d(x_{i},Y)\right),X\neq \emptyset \\ inf,X=\emptyset \end{array}\right.$$
  in which
$$d\left(y_{i},X\right)=\min_{x\in X}\left\|y_{i}-x\right\|,d\left(x_{i},Y\right)=\min_{y\in Y}\left\|x_{i}-y\right\|$$
  X and Y had the same indications in formula (3). || || was Euclidean distance. $n_{X}$ and $n_{Y}$ were the number of contour pixels in X and Y, respectively. “inf” had the same meaning in formulas ((7), (8)).
3. *N*(inf)
(10) $$N\left(inf\right)=\#\left(ASD=inf\right)$$
  in which # meant the number. ASD = inf represented that the model totally lost the ability of recognizing the target organ.
  The best segmentation corresponds to dice of 1, maxHD of 0, 95%HD of 0 and ASD of 0. 2D/3D X and Y in formulas ((3), (4), (5), (6), (7), (8), (9), (10)) correspond to 2D/3D evaluation metrics.

#### 2.4.6 Evaluation setup

The evaluations on the first and second test sets were performed in 2D and 3D, respectively.

A post‐processing of only keeping the largest 3D connected domain was conducted on the second test set. It aimed to assess the clinical effectiveness of SCOS, since such post‐processing is usually implemented in practice.

## 3 RESULTS

### 3.1 Results of SCOS‐net and its comparison with STL‐nets

**FIGURE 3:**
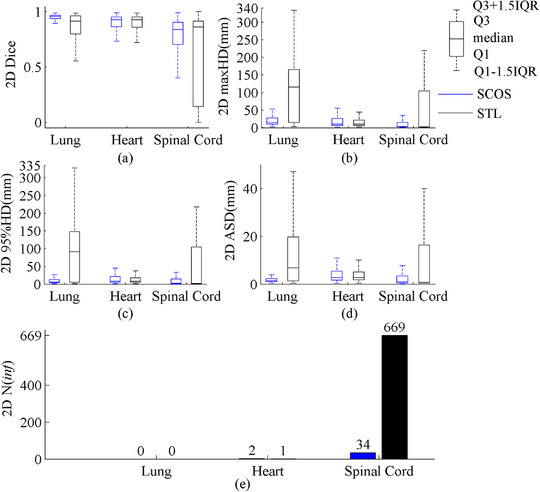
2D results of SCOS‐net and STL‐nets. STL, single‐task learning; maxHD, maximum hausdorff distance; 95%HD, 95th percentile of Hausdorff distance; ASD, average surface distance; Q1: 25th percentile; Q3, 75th percentile; IQR = Q3‐Q1; “2D” in y‐axis title: evaluation is performed on 2D slice.

The results were illustrated in the boxplot of Figure 3. SCOS‐net achieved a better segmentation for lung and spinal cord, and a comparable performance for heart with STL‐net. STL‐net showed a larger number of bad cases than SCOS‐net, since *N*(inf) = 669 for STL‐net and *N*(inf) = 34 for SCOS‐net.

### 3.2 Comparison with other multiple task settings

**FIGURE 4:**
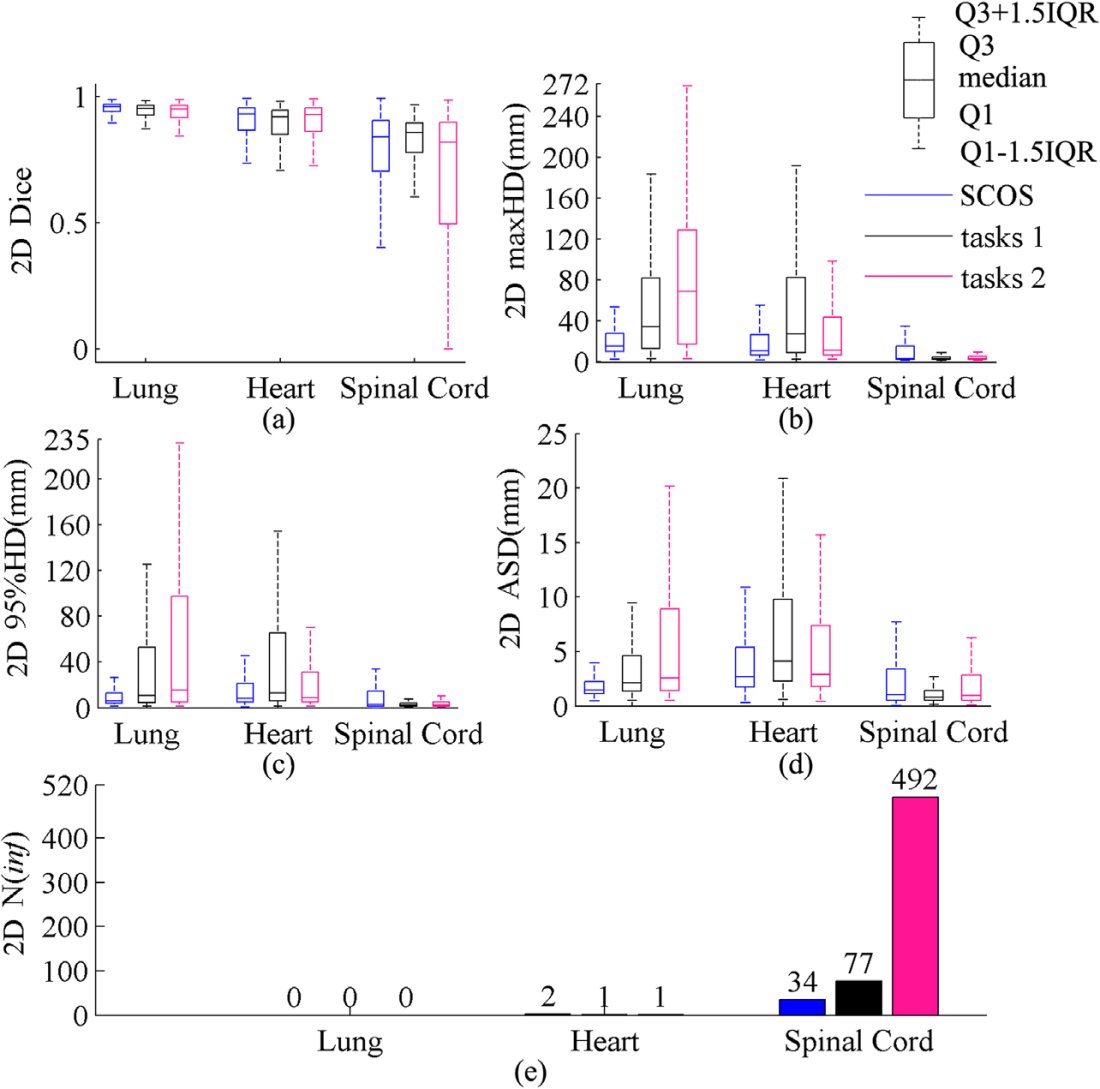
2D results of the proposed SCOS‐net and other two comparative ones. maxHD, maximum hausdorff distance; 95%HD, 95th percentile of Hausdorff distance; ASD, average surface distance; Q1: 25th percentile; Q3, 75th percentile; IQR = Q3‐Q1; “2D” in y‐axis title: evaluation is performed on 2D slice.

Figure 4 displays the comparison of different MTL settings. From this figure, SCOS‐net had the best segmentations. The spinal cord *N*(inf) of SCOS‐net is significantly smaller than two comparative settings, although its Dice and distance metrics are not the best.

### 3.3 Comparison with rotation augmentation

**FIGURE 5:**
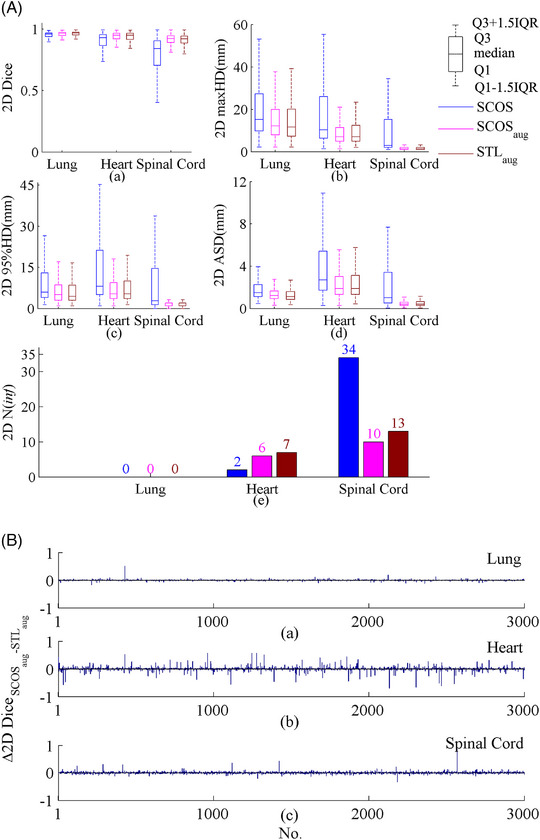
(A) 2D comparison results of SCOS‐net and rotation augmentation. (B) Bar chart of 2D Dice difference between SCOS_aug_‐net and STL_aug_‐net. In (B), (a–c) refer to lung, heart and spinal cord segmentations, respectively. The subscript “aug” means rotation augmentation. “2D” means that the evaluation is performed on 2D slice. STL, single‐task learning; maxHD, maximum hausdorff distance; 95%HD, 95th percentile of Hausdorff distance; ASD, average surface distance; Q1: 25th percentile; Q3, 75th percentile; IQR = Q3‐Q1; “2D” in y‐axis title: evaluation is performed on 2D slice.

The boxplots in Figure 5a demonstrate that the rotation augmentation indeed improves rotational robustness, since SCOS_aug_‐net and STL_aug_‐net get a better performance than SCOS‐net. The bar charts in Figure 5b are the Dice differences between SCOS_aug_‐net and STL_aug_‐net. In Figure 5b, SCOS_aug_‐net achieves better lung and spinal cord segmentations than STL_aug_‐net in some occasions, and comparable heart delineation with STL_aug_‐net.

### 3.4 Test results on real rotated cases

**FIGURE 6:**
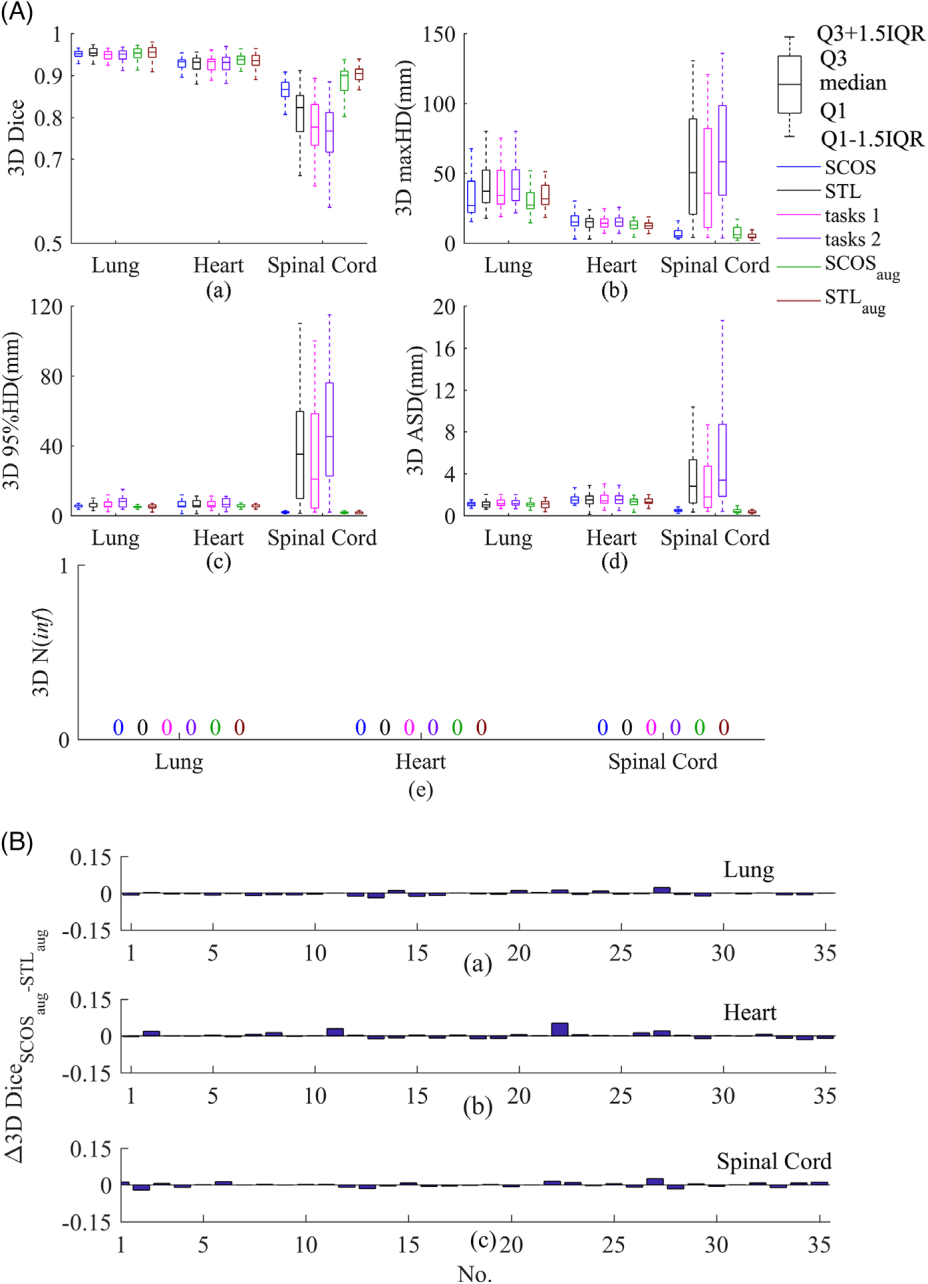
(A) 3D results of four models and rotation augmentation on real rotated cases. (B) Bar chart of 3D Dice difference between SCOS_aug_‐net and STL_aug_‐net. In (B), (a–c) refer to lung, heart, and spinal cord segmentations, respectively. The subscript “aug” means rotation augmentation. STL, single‐task learning; maxHD, maximum hausdorff distance; 95%HD, 95th percentile of Hausdorff distance; ASD, average surface distance; Q1: 25th percentile; Q3, 75th percentile; IQR = Q3‐Q1; “3D” in y‐axis title: evaluation is performed on 3D slice.

Figure 6a illustrates the 3D results on real rotated cases. In terms of no rotation augmentation, SCOS‐net shows superior. The smallest spinal cord *N*(inf) of SCOS‐net on 2D slices (shown in Figures 3 and 4) helped achieve its good spinal cord segmentation in 3D, since the post‐processing deleted those unconnected areas. With rotation augmentation, SCOS_aug_‐net and STL_aug_‐net perform better than SCOS‐net. It demonstrates the improved rotational robustness by using augmentation during model training.

**FIGURE 7:**
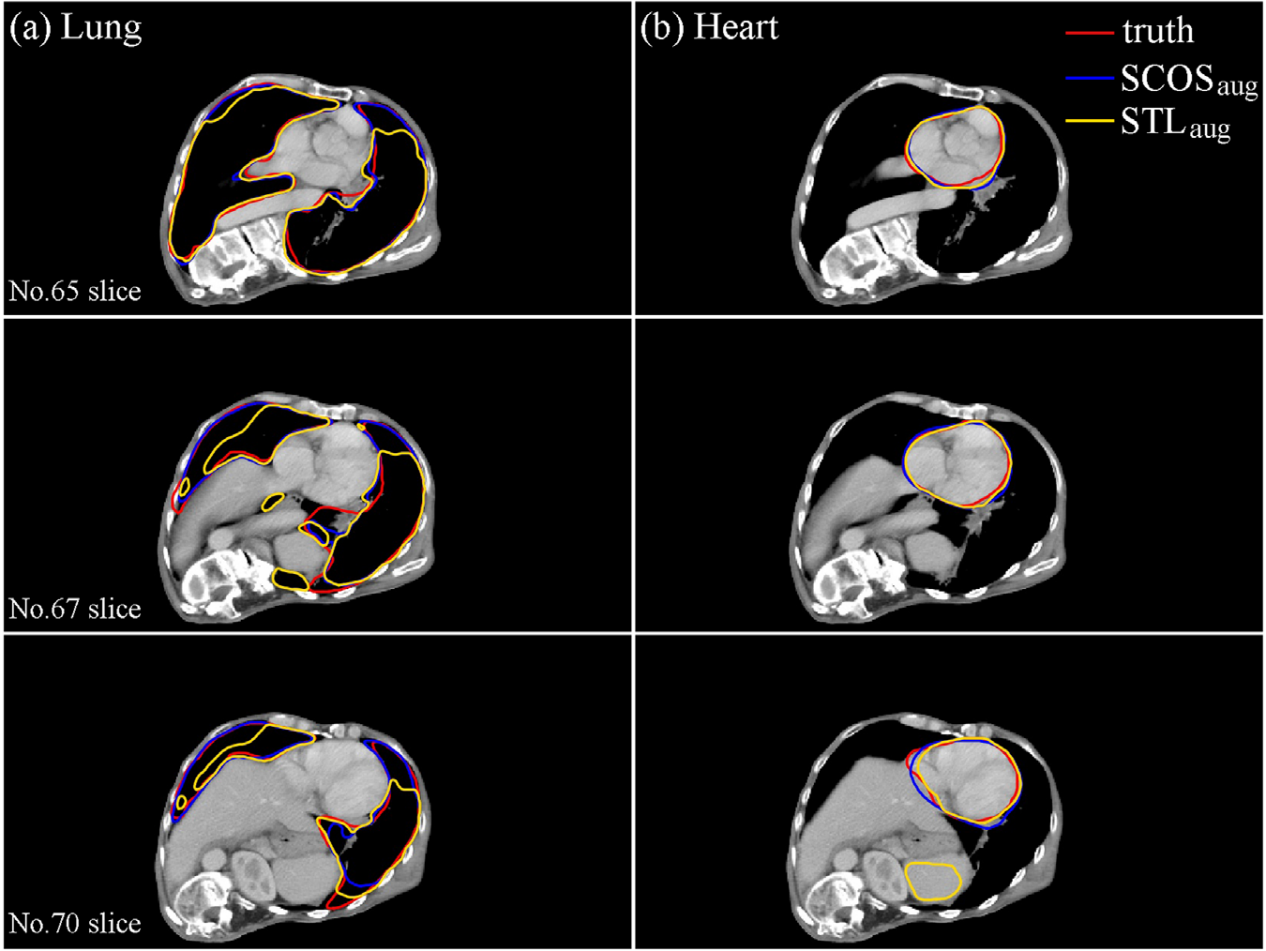
Qualitative comparison between SCOS_aug_‐net and STL_aug_‐net on an exemplary 3D rotated case. The subscript “aug” means rotation augmentation.

To further study the performance of SCOS, we plotted a bar chart (Figure 6b) to show the Dice difference between SCOS_aug_‐net and STL_aug_‐net on 3D slices owing three organs. In Figure 6b, the numbers of cases where SCOS_aug_‐net performs better/worse than STL_aug_‐net are comparable. However, in several cases, the improved Dice achieved by SCOS_aug_‐net is higher than STL_aug_‐net. Figure 7 illustrates such an example for a qualitative comparison. In Figure 7, the lung segmentations in the three slices and heart segmentations in No.70 slice suggests the superiority of SCOS.

## 4 DISCUSSION

In this section, we give a further exploration on the comparison results.

### 4.1 Discussion on the comparison between SCOS and its STL counterparts

The proposed SCOS setting aims to supplement pixel‐wise label knowledge to the local context by sharing organ‐related features in each layer. Through this way, there are two beneficial points to the model: an abundant knowledge in feature maps (1) reduces the false positive, and (2) decreases the sensitivity to the small context change, because more pixels are involved in the task. The detailed analysis is given in the following two subsections.

#### 4.1.1 Investigation on the false positive by the SCOS‐net and its STL counterparts

**FIGURE 8:**
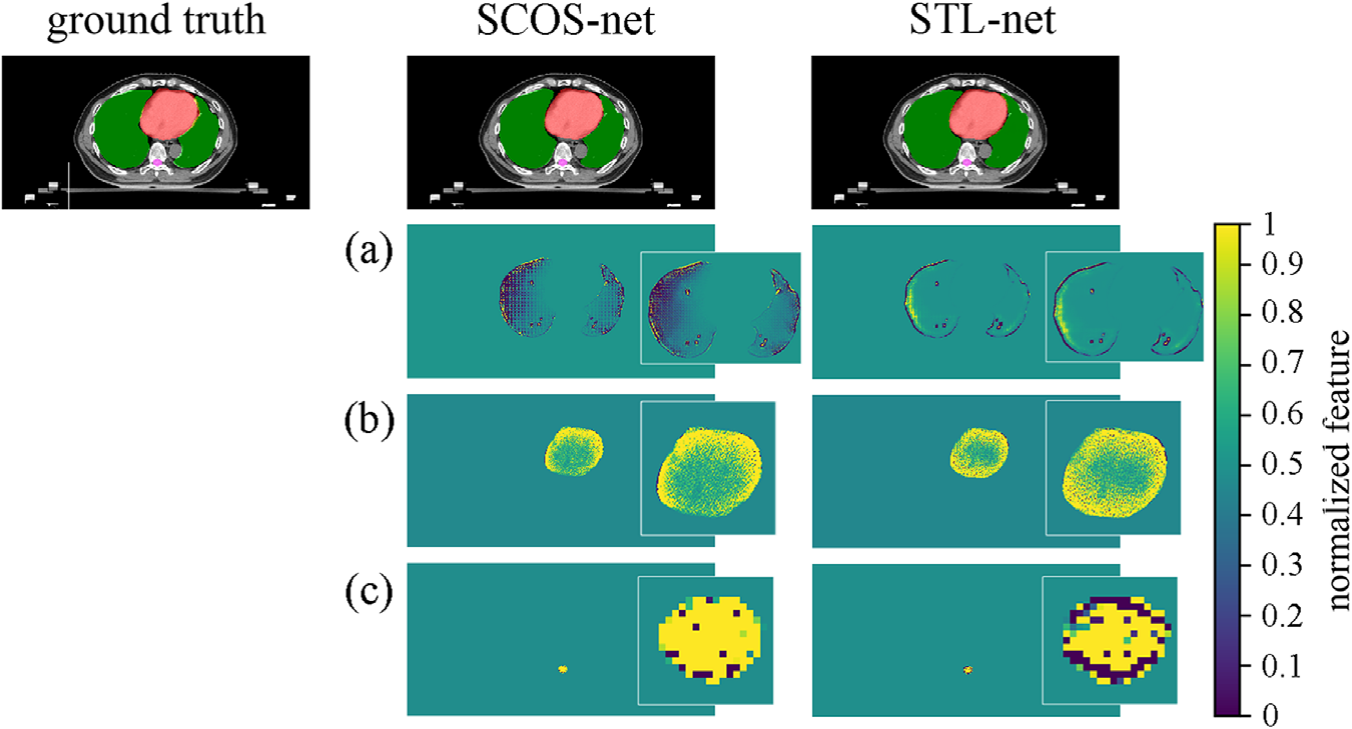
Feature maps of No.144 validating image by SCOS‐net and STL‐nets. (a‐c) are the feature maps corresponding to lung, heart, and spinal cord segmentations, respectively. In the first row, the regions of lung, heart, and spinal cord are filled by green, red, and pink separately. The feature map relates to the last convolution layer and is obtained by guided grad‐cam (i.e., a combination of guided back‐propagation and gradient class activation map). The detailed feature maps are amplified in the white boxes for a clear view.

The false positive refers to a scenario where a pixel is incorrectly labeled as target. It leads to bad distance metrics, but good Dice, just likes the lung segmentation in Figure 3. To figure the reason, we use guided grad‐cam^28^ (i.e., a combination of guided back‐propagation^29^ and gradient class activation map^28^ for FCN interpretation) to visualize the feature map of No.144 validating image in Figure 8.

**FIGURE 9:**
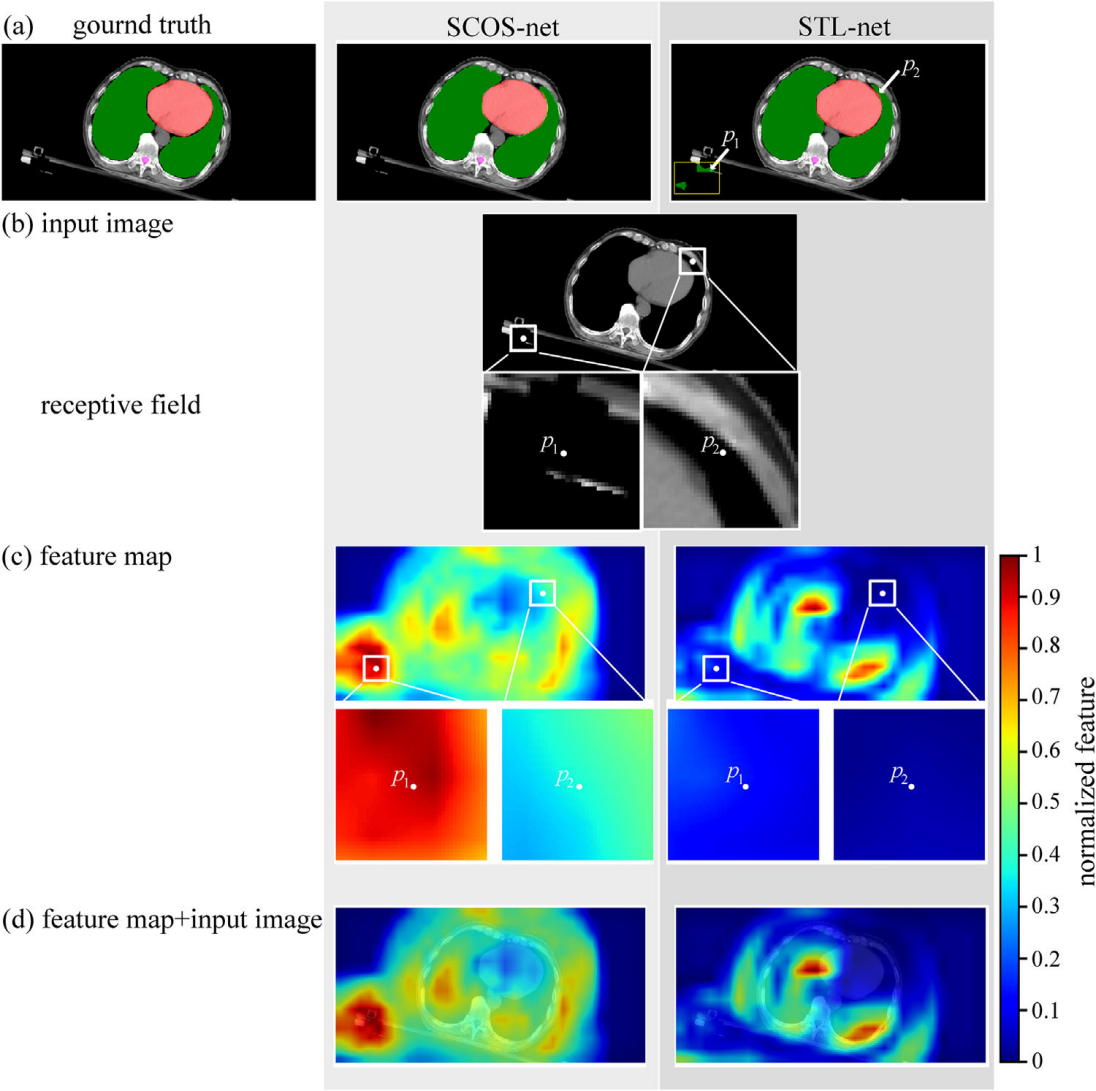
Feature map of No.1726 test image. (a) Model outputs and ground truth. The false positive is highlighted in yellow box. *p*_1_ and *p*_2_ are two comparative pixels. (b) Input image (*I*) and the receptive fields (RFs) of *p*_1_ and *p*_2_. RFs are shown in *I* using white boxes and amplified below *I*. (c) Average feature map of the last convolution layer in the encoding path. The white boxes have the same size and locations as the ones in (b). (d) Fusion of the average feature map and *I*. SCOS‐net is the proposed one. The STL‐net in this figure only refers to the lung segmentation model. In the first row, the regions of lung, heart and spinal cord are filled by green, red, and pink separately. The average feature map equals to the mean outputs of all filters and then is interpolated to the same size of *I*.

Although SCOS‐net and STL‐net both reach great match with the ground truth, their features for segmenting lung are different (Figure 8a). The pixels inside lung contribute to SCOS‐net, but STL‐net relies more on contour. It may be caused by the no knowledge provided from other organs in STL‐net. To clarify this point further, we discuss it combing with an example of false positive (i.e., No.1726 test image in Figure 9).

Figure 9a shows a false positive in segmenting lung for STL‐net. *p*_1_ belongs to the false positive. *p*_2_ is the true positive. In Figure 9B, the receptive fields (RFs) of *p*_1_ and *p*_2_ show different contexts. Part of heart is in the RF of *p*_2_. In Figure 9c, the context of *p*_2_ is distinguishable from *p*_1_ for SCOS‐net, but similar with *p*_1_ for STL‐net. By fusing feature map with the input image in Figure 9d, we find that most heart pixels correspond to zero value for STL‐net. The part of heart in the RF of *p*_2_ does not contribute to distinguishing *p*_2_ from *p*_1_. Consequently, STL‐net makes a wrong classification. For SCOS‐net, a large body of pixels show diverse feature values, and hence gives more information to the net.

#### 4.1.2 Discussion on the pixel number involved by our SCOS‐net and STL‐net

**FIGURE 10:**
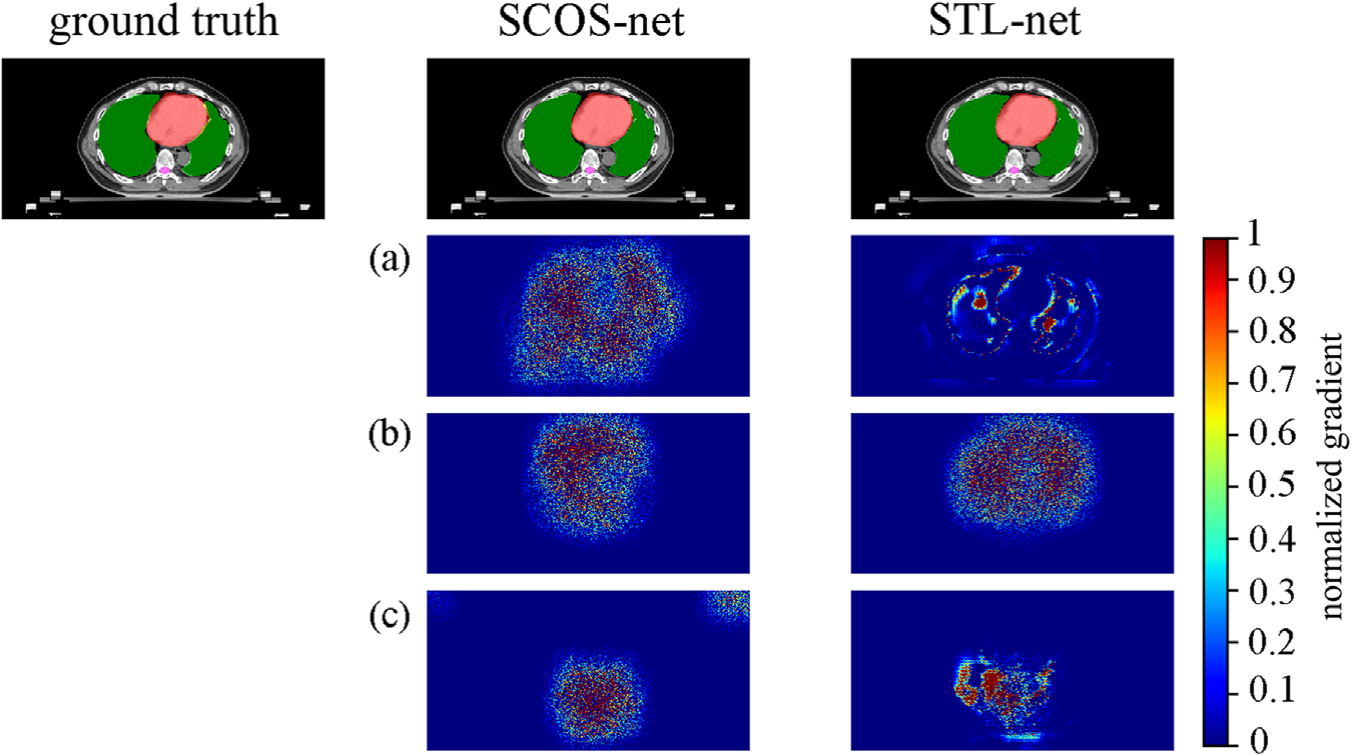
Gradient maps of No.144 validating image by SCOS‐net and STL‐nets. (a‐c) are the gradient maps corresponding to lung, heart, and spinal cord segmentations, respectively. In the first row, the regions of lung, heart, and spinal cord are filled by green, red, and pink separately.

To show how many pixels that are involved in a specified task using the proposed SCOS‐net and STL‐nets, we plotted the gradient map (***G***) of the network output with respect to its input image (detailed in Appendix) in Figure 10.

Figure 10 shows the similar segmentation results, but different ***G*** s for the two nets. In Figure 10a,c, SCOS‐net involves more pixels in segmenting lung and spinal, since the area in red for SCOS‐net is obviously larger than STL‐net. In Figure 10b, SCOS‐ and STL‐nets encompass similar size of red region. This may be the reason for the equally good heart segmentations by SCOS‐ and STL‐nets, and the superior delineations of lung and spinal cord by SCOS‐net. More knowledge involved in a task is more likely to strengthen a model's robustness.

### 4.2 Discussion on the comparison between SCOS and other two MTL settings

Figures 4 and 6a show that SCOS has a better performance on unseen rotated images than tasks 1–2. The reason may be that the spatial constraints of tasks 1–2 strengthen the network's sensitivity to the orientation of an image. When facing an unseen rotated slice, they would make a wrong classification.

### 4.3 Discussion on the comparison between SCOS and rotation augmentation

Figure 5a and 6a both demonstrate the performance of augmentation in improving rotational robustness. “SCOS+augmentation” segments better than “STL+augmentation” in some cases. It may be caused by the further exploited data of SCOS under the same condition of applying augmentation.

## 5 CONCLUSION

In this paper, we study the improved rotational robustness by using joint learning of spatially‐correlated organ segmentation. The proposed setting was implemented in a U‐net like network (abbreviated as SCOS‐net). It was validated on a large body of rotated samples, but there were no rotated ones in training set. The SCOS‐net was compared with its STL counterparts for an ablation study, and was compared with other two published multiple task settings and rotation augmentation. The results proved that the SCOS performed better than its STL counterparts and other MTL settings. “SCOS+augmentation” reached superior segmentation than “STL+augmentation” in some cases. Their performances were analyzed using visualization techniques for a better interpretation of the improved rotational robustness.

## AUTHOR CONTRIBUTIONS

All authors contributed to the study conception and design. Data collection was performed by Yiwei Yang, Min Fang, Yujin Xu, and Yongling Ji. Jie Zhang implemented the model, analyzed the results and wrote the first draft of the manuscript. Ming Chen designed the whole experiment.

## CONFLICT OF INTEREST STATEMENT

The authors declare no conflicts of interest.

## 1

The gradient map was calculated as:

(1)
$$G=\left[\begin{array}{cccccc} g_{1,1} & g_{1,2} & \cdots & g_{1,j} & \cdots & g_{1,N}\\ g_{2,1} & g_{2,2} & \cdots & g_{2,j} & \cdots & g_{2,N}\\ \vdots & \vdots & \vdots & \vdots & \vdots & \vdots \\ g_{i,1} & g_{i,2} & \cdots & g_{i,j} & \cdots & g_{i,N}\\ \vdots & \vdots & \vdots & \vdots & \vdots & \vdots \\ g_{M,1} & g_{M,2} & \cdots & g_{M,j} & \cdots & g_{M,N} \end{array}\right]$$

where ***G*** was a M×N matrix (*M* = 512, *N* = 256). The element $g_{i,j}$ was the gradient value corresponding to the input pixel in *i*th row and *j*th column. The network segmentation (***Y***) and input image (***X***) share the same size with ***G***. $g_{i,j}$ was computed as:

(2)
$$g_{i,j}=ReLU\left(\sum_{p=1}^{N}\sum_{k=1}^{M}\frac{\partial y_{k,p}}{\partial x_{i,j}}\right)$$

in which

(3)
$$ReLU\left(x\right)=\left\{\begin{array}{c} x,if\;x\geq 0\\ 0,if\;x<0 \end{array}\right.$$

$y_{k,p}$ was the element of ***Y*** in *k*th row and *p*th column. $x_{i,j}$ was the element of ***X*** in *i*th row and *j*th column. With ReLU, ***G*** only shows the pixels which have positive effects on output. The larger $g_{i,j}$ indicates the greater impact on network output.
